# Discrimination of rippled-spectrum patterns in noise: A manifestation of compressive nonlinearity

**DOI:** 10.1371/journal.pone.0174685

**Published:** 2017-03-27

**Authors:** Olga N. Milekhina, Dmitry I. Nechaev, Vladimir O. Klishin, Alexander Ya. Supin

**Affiliations:** Institute of Ecology and Evolution, Russian Academy of Sciences. 33 Leninsky Prospect, Moscow, Russia; Institute of Deep-sea Science and Engineering, Chinese Academy of Sciences, CHINA

## Abstract

In normal-hearing listeners, rippled-spectrum discrimination was psychophysically investigated in both silence and with a simultaneous masker background using the following two paradigms: measuring the ripple density resolution with the phase-reversal test and measuring the ripple-shift threshold with the ripple-shift test. The 0.5-oct wide signal was centered on 2 kHz, the signal levels were 50 and 80 dB SPL, and the masker levels varied from 30 to 100 dB SPL. The baseline ripple density resolutions were 8.7 oct^-1^ and 8.6 oct^-1^ for the 50-dB and 80-dB signals, respectively. The baseline ripple shift thresholds were 0.015 oct and 0.018 oct for the 50-dB and 80-dB signals, respectively. The maskers were 0.5-oct noises centered on 2 kHz (on-frequency) or 0.75 to 1.25 oct below the signal (off-frequency maskers). The effects of the maskers were as follows: (i) both on- and low-frequency maskers reduced the ripple density resolution and increased the ripple shift thresholds, (ii) the masker levels at threshold (the ripple density resolution decrease down to 3 oct^–1^ or ripple shift threshold increased up to 0.1 oct) increased with increasing frequency spacing between the signal and masker, (iii) the masker levels at threshold were higher for the 80-dB signal than for the 50-dB signal, and (iv) the difference between the masker levels at threshold for the 50-dB and 80-dB signals decreased with increasing frequency spacing between the masker and signal. Within the 30-dB (from 50 to 80 dB SPL) signal level, the growth of the masker level at threshold was 27.8 dB for the on-frequency masker and 9 dB for the low-frequency masker. It is assumed that the difference between the on- and low-frequency masking of the rippled-spectrum discrimination reflects the cochlear compressive non-linearity. With this assumption, the compression was 0.3 dB/dB.

## Introduction

The ability to discriminate spectral patterns (spectral resolution) is an important characteristic of the auditory system. To a significant extent, it determines the ability to discriminate complex acoustic signals. The majority of existing methods for measuring spectral resolution in humans are based on the assessment of the frequency tuning of the auditory filters using masking techniques. A variety of masker types were used in those investigations as follows: pure tone or narrowband maskers (the tuning curve paradigm; see [1] for a review); two-tone maskers [2–4]; narrow-band noise (the critical band paradigm; see [5] for a review); notch-noise [6–8]; or rippled noise [4, 9–12].

In these investigations, the basilar membrane was considered a bank of equivalent frequency-tuned auditory filters with certain qualities. Several analytical expressions have been suggested to summarize the data on the equivalent rectangular bandwidth (ERB) of the auditory frequency-tuned filters as a function of the center frequency, e.g., [13]. Based on this (or a similar) equation and ignoring possible nonlinear interactions between the frequency-tuned filters, the responses of the basilar membrane to signals with various spectral compositions may be predicted.

The prediction of the response to complex-spectrum signals may be more difficult in cases when sounds appear in a background of other sounds, which may be considered noise for a certain target signal. To investigate the influence of background noise on the frequency resolution, masking methods involve the use of the masking background together with another masker for the frequency-tuning measurements, which may result in complicated interactions that are difficult to evaluate. However, several other experimental paradigms have revealed a deterioration of the frequency discrimination in the noise background.

In particular, the influence of noise on frequency discrimination limens has been demonstrated. Frequency discrimination limens for pure tones do not directly measure frequency tuning of the auditory filters; they are less than 1% of the center frequency [14–18], whereas the filter bandwidths are approximately 10% of the center frequency. This difference between the frequency discrimination limens and critical band width may appear due to detection of changes in the excitation level at the steep slopes of the excitation pattern produced by the pure tone [17, 19–21] or due to extraction of the frequency-shift information from the temporal patterning (phase locking) of the neural responses [22, 23]. However, frequency discrimination limens may be associated with frequency tuning: the better the tuning, the smaller the limens. It has been shown that masking noise impairs pure-tone frequency discrimination. Frequency discrimination worsens with a decreasing signal/noise ratio [19]. Experiments using notched noise have shown that on-frequency and low-frequency noise impairs frequency discrimination [24–26]. The issue was considered in a previous study [27].

Another paradigm for measuring frequency tuning is based on a direct estimation of the spectrum pattern resolution [28–30]. These methods have used rippled noise (a spectrum “grid”) as the probe sound; this signal has a spectrum with periodically alternating peaks and troughs. This approach is close to the spectrum profile analysis [31, 32]. Considering that many natural sounds have wider and more complicated frequency spectra than pure tones, the rippled-spectrum signals were taken as representatives of such multicomponent signals. However, unlike many natural signals, their spectral structure that can be precisely described by a limited number of parameters. In several studies [29, 30, 33], a ripple phase-reversal test was exploited to find the highest resolvable ripple density. In other studies [34, 35], a ripple phase shift was exploited to find the ripple-shift threshold. Contrary to masking-based methods, these tests directly indicate whether the fine spectrum pattern of the rippled-spectrum stimulus is or is not discriminated. Using this method, the rippled-spectrum resolution was investigated in background noise. Those studies demonstrated that noise maskers impaired the spectrum-pattern resolution in terms of the ripple density [36–38] and ripple shift [35] when the masker band coincided with the rippled-spectrum signal (on-frequency maskers) or when it was lower in frequency than the signal (low-frequency maskers).

It is likely that the effect of maskers on the spectrum-pattern resolution depends at least (but not only) on the signal-to-masker ratio, signal and masker SPL, and the frequency spacing between the signal and masker. In the abovementioned studies, only certain aspects of this complex dependence have been investigated. Complicated nonlinear interactions between various components of the signal and masker make it difficult to predict the spectrum-pattern resolution for a particular combination of these components. Therefore, a direct investigation of the influence of several signal and masker parameters on spectrum resolution may be helpful.

The present study is an attempt to investigate the discrimination of spectrum patterns over a range of combinations of signal and masker parameters. The goal of this attempt was to assess, at least for a specific spectrum pattern, the parameters of masking noise that do prevent or do not prevent discrimination of the pattern. Assuming that the frequency and level are primary characteristics of sounds, the variable parameters in the present study were the signal level, masker level (and, therefore, the signal/masker ratio), and signal/masker frequency spacing.

We considered that the spectrum-pattern resolution may be assessed in different manners. In particular, it may be assessed in the following ways: (i) by the finest resolvable spectrum pattern, which may be represented by the ripple density and (ii) by the least detectable modification of the spectrum, which may be represented by ripple shift. Therefore, we exploited the following two tests of rippled-spectrum discrimination: (i) measurement of the ripple density resolution using the phase-reversal test and (ii) measurement of the ripple-shift threshold using the ripple-shift test.

## Materials and methods

### Listeners

Eight listeners (four males and four females, laboratory staff and volunteers) participated in this study. The listeners were 23–63 years old and had hearing thresholds of less than 15 dB over the range of 1–4 kHz, where the signals and maskers were presented. All listeners signed informed consent forms to participate in the experiments. All listeners had experience with psychoacoustic experiments with complex-spectra discrimination tasks.

The experiment and consent procedures were approved by the Ethics Committee of the Institute of Ecology and Evolution, Russian Academy of Sciences, where the study was performed for sounds of SPLs ≤ 100 dB and everyday sound exposure levels (SEL) ≤ 120 dB in compliance with National Sanitary Normative SN2.2.4/2.1.8.562–96.

### Signals and maskers

**Fig 1:**
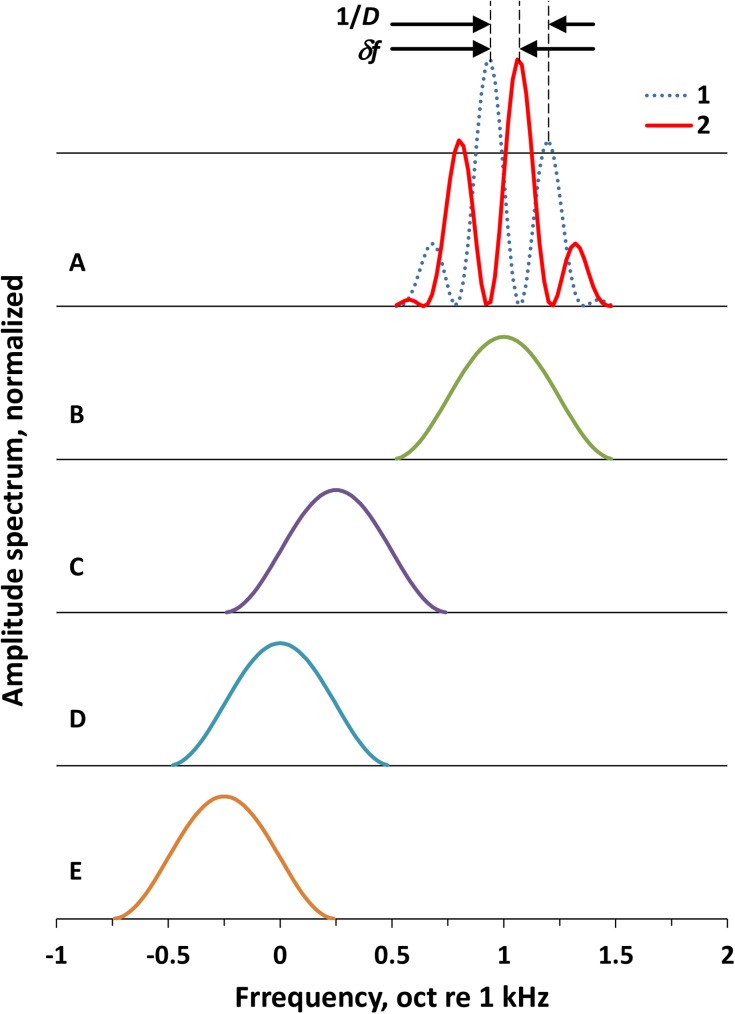
Frequency spectra of the signal and maskers. **A.** Signal. **B.** On-frequency masker. **C.** Low-frequency masker, 0.75 oct below the signal. **D.** Low-frequency masker, 1 oct below the signal. **E**. Low-frequency masker, 1.25 oct below the signal. In A: 1 and 2 –filter forms for spectra replacing one another in the ripple-shift or ripple reversal test, 1/*D*–ripple spacing (*D*–ripple density), and *δf*–ripple shift.

The test signals were band-limited rippled noises (Fig 1). The envelope of the spectrum was a one-octave wide cycle of a cosine function of log frequency that was centered at 2 kHz (1 oct re 1 kHz, Fig 1A). According to previous studies, this center frequency was characterized by a high resolution of both the ripple density [29, 30] and ripple shift [34]. The cosine envelope was used to avoid the effects of sharp spectrum edges, which might influence the resolution of the ripple patterns [38].

The spectrum form was a product of this envelope and ripples that were defined by a sine function of the log frequency. These ripples appear uniformly spaced on the log frequency scale, as shown in Fig 1A. For this type of ripple pattern, a convenient metric of its ripple density, *D*, is the number of ripples per octave (RPO), oct^–1^. The frequency spacing between the adjacent ripples is 1/*D* (oct).

For measurements, two signal types were exploited, the test and reference signals (see the Experimental Procedure below). The test signal was a rippled-spectrum noise burst lasting 2.4 s. During this burst, every 0.4 s, one of the rippled spectra presented in Fig 1A was replaced with another spectrum with a position of ripples on the frequency scale differing by *δf* and back; the two spectra were alternatively presented during the signal. Each test signal contained six ripple shifts (three up/down cycles of shifts). The 0.4-s presentation of each of the signal versions was used because, at shorter durations, the difference between the rippled-spectrum versions could not be successfully extracted from fluctuations intrinsic to the rippled noise [30].

The reference signal was a rippled-spectrum noise burst lasting 2.4 s with the same ripple density and level as the test signal but without the ripple shifts (either spectrum 1 or 2, as presented in Fig 1A, randomly chosen). On average, the root-mean-square (RMS) level of this signal was -13 dB relative the highest ripple peak; its level-weighted mean frequency was 2.03 kHz and equivalent rectangular bandwidth (ERB) was 0.28 oct.

**Fig 2:**
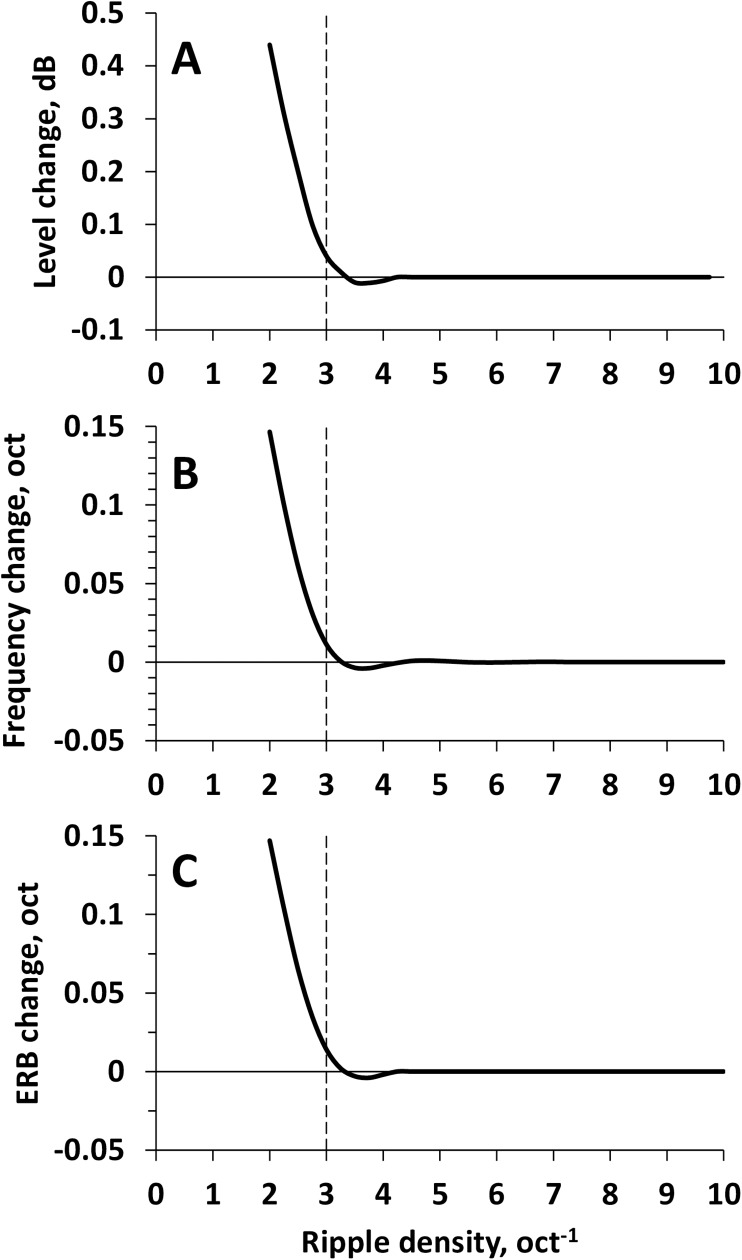
Changes of signal parameters at ripple phase reversal as functions of the ripple density. **A.** Level. **B.** Center frequency. **C.** ERB. All the changes are minor at ripple densities of 3 oct^-1^ and higher.

When ripples were shifted, the level, center frequency, and ERB of the signal changed due to different interrelations of the ripples and spectrum envelope. These changes decreased with increasing ripple density. At ripple densities above 3 oct^-1^, the level changes did not exceed 0.04 dB (Fig 2A), mean frequency changes did not exceed 0.01 oct (Fig 2B), and ERB changes did not exceed 0.015 oct (Fig 2C), even for the largest possible ripple shift (reversal). We considered these changes negligible because they were less than random fluctuations in the level, center frequency, and ERB (see below).

**Fig 3:**
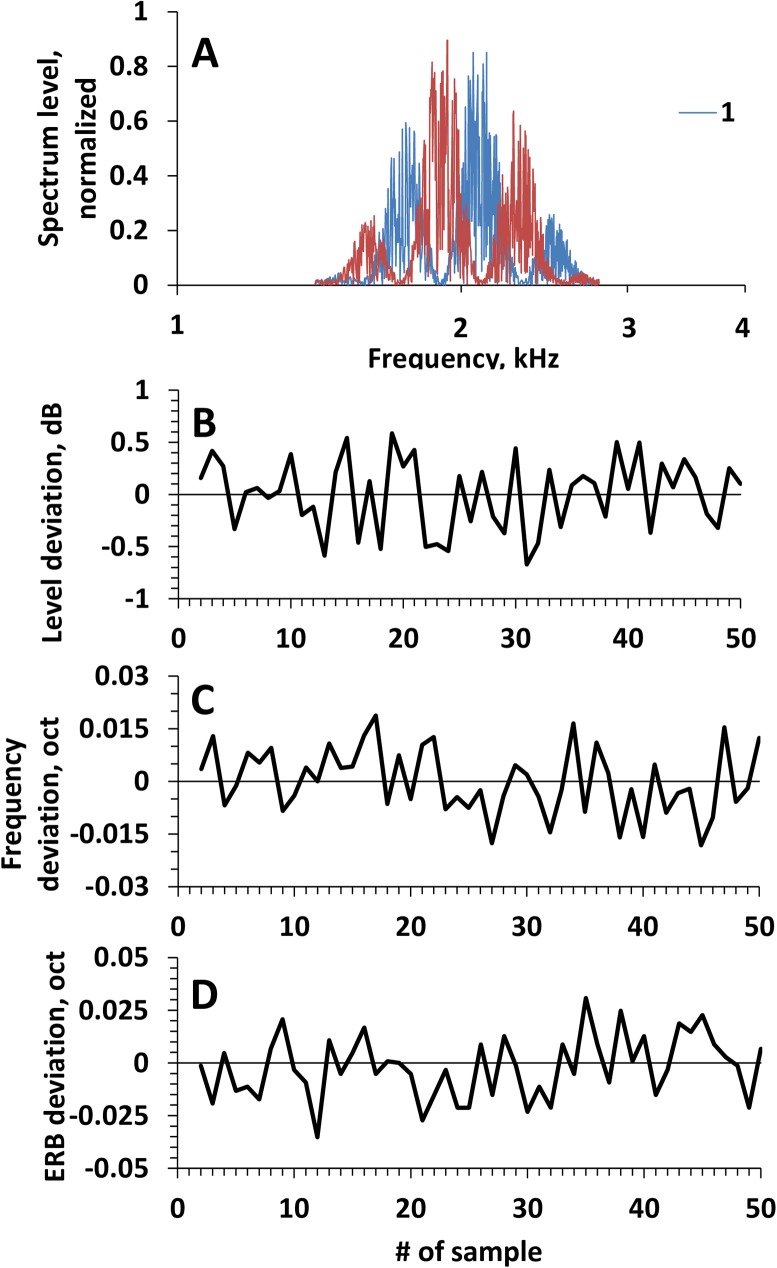
Signal random fluctuations. **A.** Spectra of arbitrarily chosen 400-ms long rippled noise bursts (ripple density of 3.5 oct^-1^) with opposite ripple phases. Spectra 1 and 2 satisfactorily reproduced spectrum shapes 1 and 2 in Fig 1A. **B.** Variation in the RMS levels of 50 randomly chosen 400-ms rippled-noise bursts (ripple density of 3.5 oct^-1^); deviations in the levels from the averaged level are within ±0.7 dB. **C.** Variation of the center frequencies of 50 randomly chosen 400-ms rippled-noise bursts (ripple density of 3.5 oct^-1^); deviations are within ±0.02 oct. **D.** Variation of the ERB of 50 randomly chosen 400-ms rippled-noise bursts (ripple density of 3.5 oct^-1^); deviations are within ±0.03 oct.

Spectra of rippled noise bursts of a limited duration (0.4 s presentation of each rippled spectrum version) did not precisely reproduce the predefined spectrum forms as those presented in Fig 1 because of fluctuations intrinsic in the noise. However, the spectra of noise bursts satisfactorily reproduced the spectrum envelope and rippled pattern (Fig 3A).

Because of noise fluctuations, the signal level of individual bursts fluctuated within less than ±0.7 dB (standard deviation, SD = 0.34 dB; Fig 2B), the center frequency fluctuated within less than ±0.02 oct (SD = 0.01 oct; Fig 2C), and ERB fluctuated within less than ±0.03 oct (SD = 0.015 oct).

Signal levels were fixed at either 50 or 80 dB SPL. Signal levels below 50 dB SPL were not used because signals of 40 dB SPL or lower resulted in worse sound-pattern discrimination [27]. Signal levels above 80 dB SPL were not used because, at signal levels of 90 dB SPL and higher, off-frequency masking appeared at masker levels above the allowed level limit of 100 dB.

The maskers were band-limited noises with a spectrum that was a one-octave wide cycle of a cosine function of log frequency (Fig 1B–1E). The spectrum was centered either on the same 2 kHz frequency as the signal (the on-frequency masker, Fig 1B) or at 1.19, 1.0, or 0.84 kHz, i.e., 0.75, 1, or 1.25 octaves below the signal, respectively (low-frequency maskers, Fig 1C–1E). Maskers were presented as 2.4-s long bursts simultaneously with both the test and reference signals (simultaneous masking).

### Signal and masker generation

The signals and maskers were digitally generated at a sampling rate of 32 kHz. For the signal generation, a wideband signal (a random digital sequence) was digitally filtered by one of the filters with the frequency responses presented in Fig 1A. For the generation of a test signal, every 0.4 s, the wideband signal was redirected from one of the filters presented in Fig 1A to the other filter and back; six switches (three up-down switch cycles) resulted in a 2.4-s signal duration. To generate a reference signal, only one of these two filters was used (either 1 or 2, randomly alternating trial-by-trial) during the 2.4 s. For the generation of a masker burst, one of the filters presented in Fig 1B–1E was used for 2.4 s. The signal and masker were summed. Details of the generation routine have been described earlier [34].

Noteworthy features of the generation routine were as follows:

1. For the signal and masker generation, the noise burst onset and offset, as well as the phase shifts, were performed by switching the wideband signal at the inputs of the filters rather than switching the outputs of the filters. Therefore, the transitions from one spectrum to the other, as well as the onset/offset ramps, were determined by the filter transfer functions, and the signal spectrum never exceeded the filter passband, i.e., no wideband transients occurred at the phase shifts. Additionally, at the ripple phase shifts, there was no increase or decrease in the signal root-mean-square (RMS) that exceeded the fluctuations intrinsic in the noise. With this generation technique, the switches produced no noise waveform envelope fluctuations that exceed background fluctuations intrinsic in noise (Fig 2).
2. The signals and maskers were generated using different sources of the wide-band signal, i.e., they were not coherent. Therefore, the overall signal + masker presented a sum of the signal and masker powers rather than the amplitudes.
3. In two alternative spectra (1 and 2 in Fig 1), the largest ripples were symmetrically positioned relative the envelope peak. This condition minimized the change in the signal level at the transition from one spectrum to the other.

### Experimental procedure

Two versions of measurement were used: to measure the ripple density resolution and to measure the ripple shift threshold. For both versions of the measurements, a two-alternative forced-choice procedure with feedback was used. The following two signals were presented in each trial: the test and reference signals. Each signal lasted 2.4 s with a 0.4-s interval between signals. The order of signal presentation (first test, second reference, or vice versa) varied randomly, trial-by-trial. In the control (no masker) runs, only the test and reference signals were presented. In the masking runs, maskers were played simultaneously with both the test and reference signals, each lasing 2.4 s. The listener was asked to detect any periodic modifications in the played sound that occurred every 0.4 s and to report which of the signals in the pair (the first or second) featured periodic (every 0.4 s) modifications, i.e., to detect the test signal. The listener was informed of whether the response was correct or incorrect (feedback).

For the ripple-density resolution measurements, the ripple shift in the test signal was maintained constant at 1/2*D*, i.e., at the shift, the ripple phase was reversed and the peaks and troughs of the ripples were interchanged. The ripple density *D* was varied, trial-by-trial, between the following values: 2, 2.5, 3, 3.5, 4, 5, 6, 7, 8, 10, and 12 oct^-1^. The density of 2 oct^–1^ was the lowest available at the signal ERB of 0.5 oct because the ripple structure disappeared at lower densities. The variation was performed in an adaptive manner. After three correct detections of the test signal, the ripple density in the next trial was increased by one step (i.e., ripple spacing decreased). Following every incorrect detection of the test signal (a “mistake”), the ripple density was decreased by one step (ripple spacing increased) (a one-up, three-down version of the adaptive procedure).

For the ripple-shift threshold measurements, the ripple density in the test signal was maintained constant at 3.5 oct^-1^. This ripple density has been shown to provide the lowest baseline threshold due to optimal combination of the ripple spacing and ripple ramp gradient [34]. The ripple shift was varied, trial-by-trial, by an adaptive procedure. After three correct detections of the test signal, the ripple shift in the next trial was decreased by 0.005 oct; after every mistake, it was increased by the same step (a one-up, three-down version). At a ripple density of 3.5 oct^-1^, the maximum available ripple shift was 0.14 oct because larger shifts were equal to the lesser shifts in the opposite direction.

Every run was initiated with a ripple spacing or shift that was well above the anticipated threshold. The part of the run before the first mistake was considered a “warming-up” segment. The part of the run after the warming-up segment was considered a measurement segment. The procedure lasted until eight reversals occurred. The ripple density or ripple shift values at the eight reversals were averaged. The result was adopted as an estimate of the ripple density resolution or ripple shift threshold for this particular run. If the ripple density was not resolvable at the lowest available density of 2 oct^–1^, a resolution of 1 oct^–1^ was arbitrarily ascribed to this run. If the ripple shift threshold was not detectable at the highest available shift of 0.14 oct, a threshold of 0.28 oct was arbitrarily ascribed to this run. For each combination of signal and masker parameters, the measurements were repeated three times for each of the eight listeners. The results of the 24 measurements (three measurements in each of 8 listeners) for every combination of the signal and masker parameters were averaged to yield a final density resolution or shift threshold estimate as a mean with a standard error (SE).

### Instrumentation

The signals and maskers were digitally synthesized on a standard personal computer using a custom-written program (virtual instrument) designed with LabVIEW software (National Instruments, Austin, TX, USA). The digitally generated signals were digital/analog (D/A) converted using a 16-bit D/A converter in a data acquisition board NI USB-6251 (National Instruments). The analog signals were power amplified, attenuated, and diotically played through HD580 headphones (Sennheiser, Wedemark, Germany). The frequency response of the headphones varied by no more than 1.5 dB within 0.5-octave bands of the signal and maskers, which was measured using a Testo 816 noise level meter (Testo AG, Lenzkirch, Germany) equipped with a 0.5-in microphone that terminated through a 6-cm^3^ coupler. The sound level of the signals was measured in the same manner. The listener sat in a sound-attenuating booth, MINI 350 (IAC, Germany).

## Results

### Baseline thresholds

The baseline (no masker) ripple density resolution and ripple shift thresholds were measured for signal levels of 50 and 80 dB SPL. For the ripple density, the baseline resolution estimates (means ± SE) were 8.7 ± 0.5 oct^-1^ for the 50-dB signal and 8.6 ± 0.3 oct^-1^ for the 80-dB signal. These thresholds did not significantly differ from one another (*p* = 0.93 by paired *t*-test).

For the ripple shifts, the baseline threshold estimates were 0.015 ± 0.002 oct for the 50-dB signal and 0.018 ± 0.002 oct for the 80-dB signal. The difference of 0.003 oct was not statistically significant (*p* = 0.27).

### Masked thresholds

**Fig 4:**
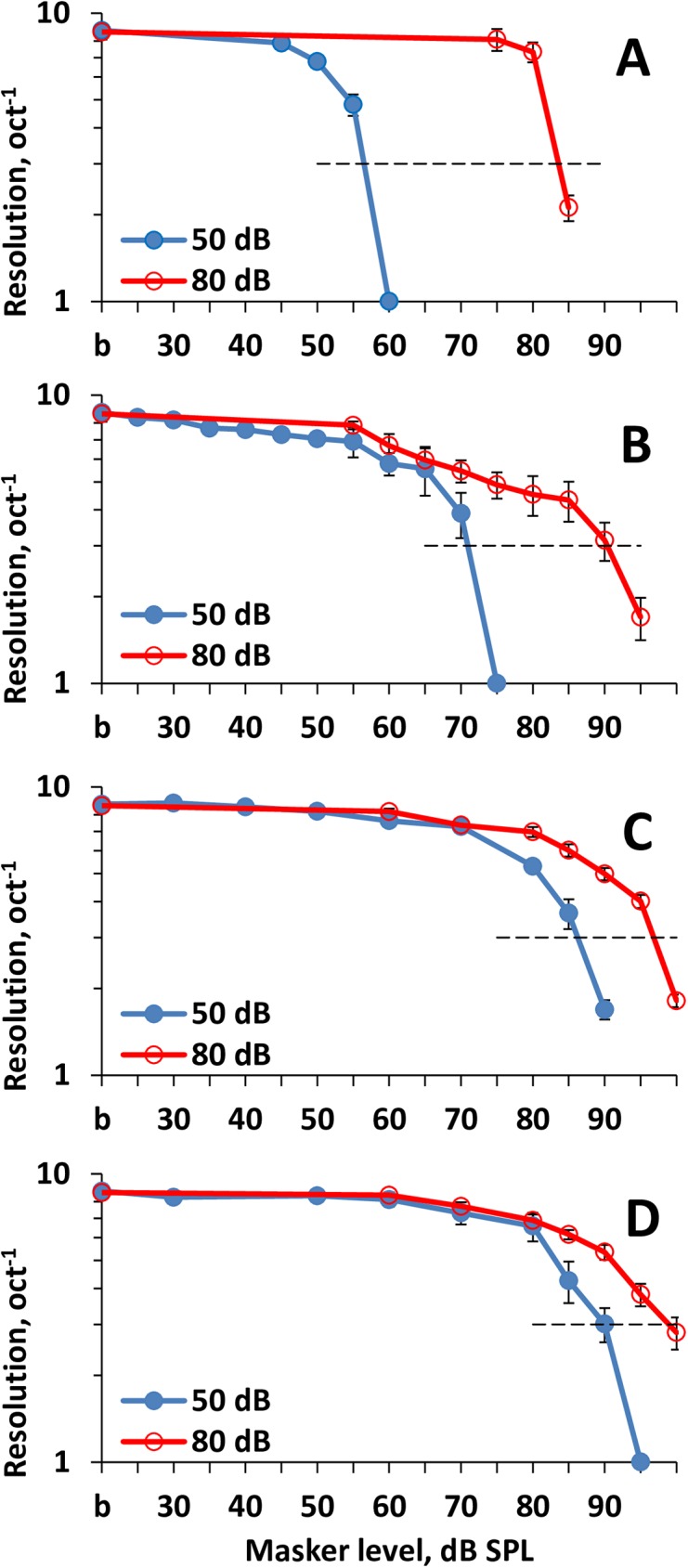
Ripple density resolution dependence on masker SPL. **A:** On-frequency masker. **B-D:** Off-frequency maskers centered at 0.75, 1.0, and 1.25 oct below the signal, respectively. Resolutions are presented for two signal levels (50 and 80 dB SPL, as indicated in the legend). *b*–baseline (non-masked) resolution. Horizontal dashed lines at a level of 3 oct –^1^ –the level of evaluation of plots shift relative to one another. Error bars–standard errors.

**Fig 5:**
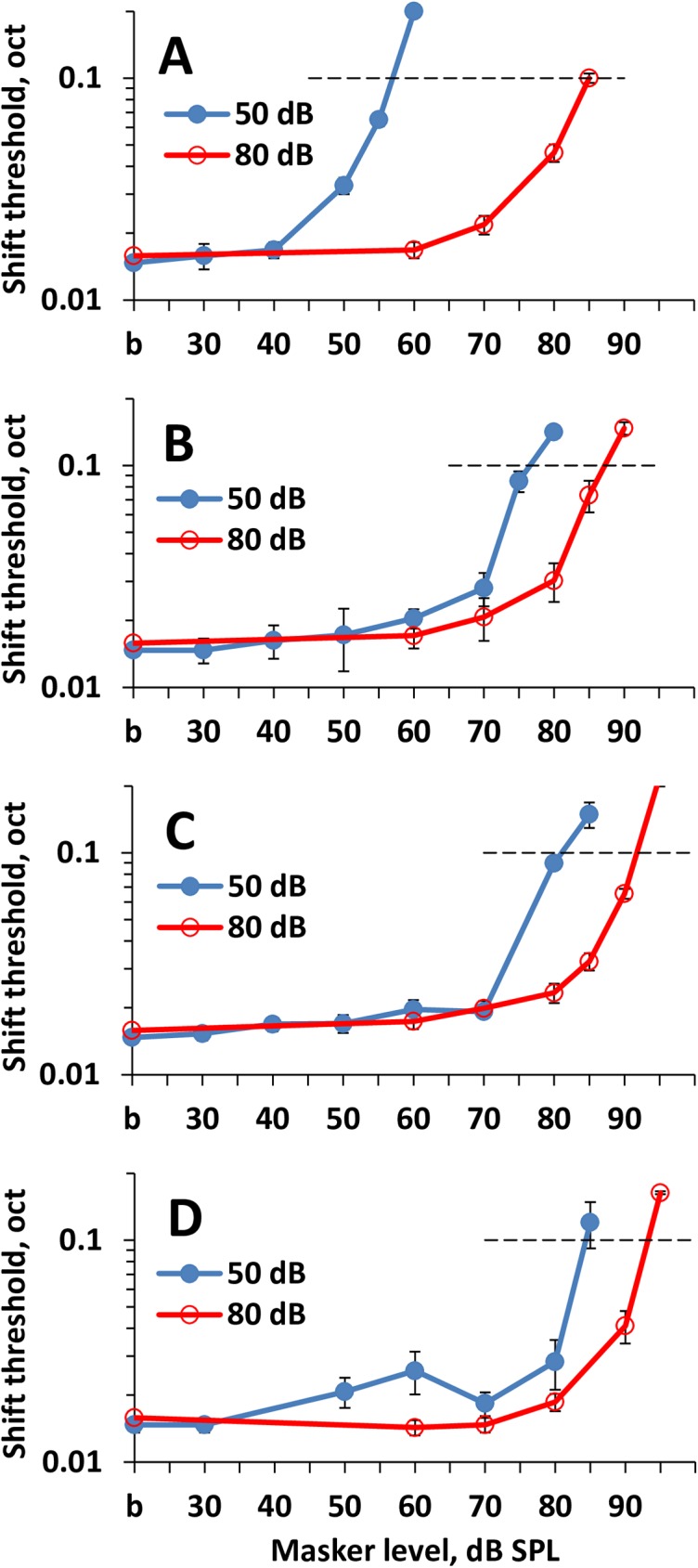
Ripple shift threshold dependence on masker SPL. **A:** On-frequency masker. **B-D:** Off-frequency maskers centered at 0.75, 1.0, and 1.25 oct below the signal, respectively. Thresholds are presented for two signal levels (50 and 80 dB SPL, as indicated in the legend). *b*–baseline (non-masked) threshold. Horizontal dashed lines at a level of 0.1 oct–the level of evaluation of plots shift relative to one another. Error bars–standard errors.

The masked ripple density resolution and ripple shift thresholds were measured for signal levels of 50 and 80 dB SPL and masker levels from 30 to 100 dB SPL. The results of the measurements are presented in Figs 4 and 5 and can be summarized as follows.

1. An increase in the masker level decreased discrimination of the rippled pattern of the signals. It manifested both in a decrease in the ripple density resolution (Fig 4) and an increase in the ripple shift thresholds (Fig 5).
2. By increasing the frequency spacing between the signal and masker, higher masker levels were required to obtain a certain masking effect as demonstrated by the plots shown in A to D in both Fig 4 and Fig 5.
3. Obtaining a certain masking effect for the 80-dB signal required a higher masker level than that for the 50-dB signal.
4. The difference between the masker levels required for equal effects on 50-dB and 80-dB signals depended on the frequency spacing between the signal and masker as follows: The greater the frequency spacing, the lower the difference between the equally effective masker levels for 50-dB and 80-dB signals.

We attempted to quantitatively characterize the difference between the masker levels required for a certain masking effect on 50-dB and 80-dB signals. We evaluated the masker levels that reduced the ripple density resolution to an arbitrarily chosen value of 3 oct^–1^ and a ripple shift threshold to an arbitrarily chosen value of 0.1 oct. These arbitrary criterion levels were chosen because, for the majority of the plots, these levels were close to the last measurable values. An additional point to emphasize was that at ripple densities of 3 oct^-1^ and higher, the changes in the signal level, mean frequency, and ERB were negligible at ripple shifts (see methods). The dB-distances between the plots for the 50-dB and 80-dB signals at the specified criterion levels were taken as the shifts of the masking effect-vs-masker level functions relative to one another along the masker level scale.

**Fig 6:**
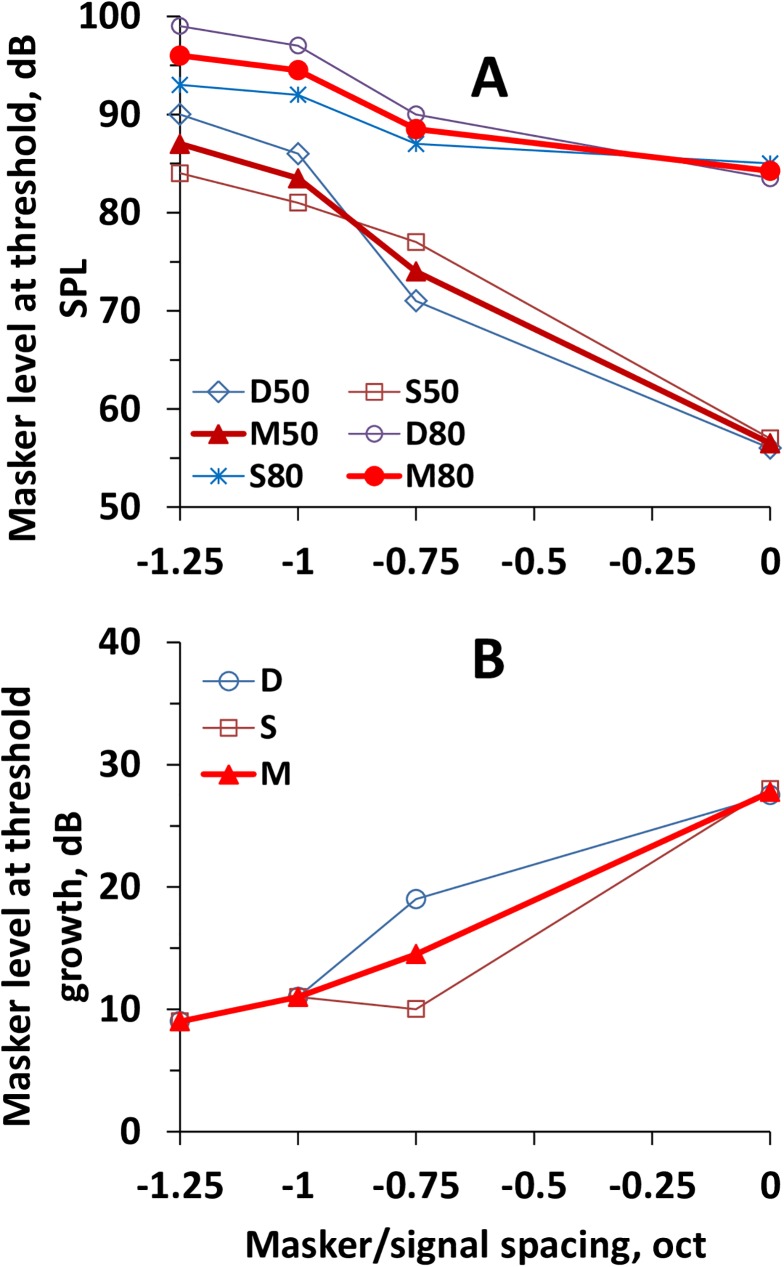
**Dependence of the masker SPL at threshold (A) and difference for 50-dB and 80-dB signals (B) on the masker frequency.** The masker frequency is presented in octaves relative to the probe frequency. In **A**: D50, S50, and M50 –data obtained by the ripple density resolution (D50), ripple shift threshold (S50), and mean of the two (M50) for the 50-dB signal; D80, S80, and M80 –the same for the 80-dB signal. In **B**: D, S, and M–data obtained by the ripple density resolution, ripple shift threshold, and mean of the two, respectively.

The results of the measurements are presented in Fig 6A for both the ripple density resolution and ripple shift threshold. Both tests revealed similar results. For the on-frequency masker (0-oct masker/signal frequency spacing in Fig 6A), the masker levels at threshold (means of the ripple density resolution and ripple shift threshold data) were 56.5 dB SPL for the 50-dB signal and 84.3 dB for the 80-dB signal. Therefore, the difference between the masker levels at threshold could be evaluated as 27.8 dB (Fig 6B), i.e., close to the 30-dB difference between signals. With increasing masker/signal frequency spacing, the difference between the masker levels at threshold decreased. For a masker of –1.25 oct, which may be considered as a truly off-frequency masker, the masker level at threshold was 87 dB for the 50-dB signal and 96 dB for the 80-dB signal. Therefore, for the low-frequency maskers, the difference between the masker levels at threshold was as small as 9 dB. For the low-frequency maskers of –0.75 and –1 oct, the masker level differences were intermediate between those for the on- and low-frequency maskers.

## Discussion

It has previously been demonstrated for frequency discrimination limens that the frequency resolution is poorer in noise than in quiet conditions [19, 24–26]. However, the net effect of background noise on the resolution of complex spectra is difficult to predict from those data because of the complicated dependence of this effect on the frequency and level of spectral components. The present study directly demonstrated the deteriorating effect of the background noise, at least for a specific version of complex spectra. The data quantitatively show the parameters at which a masker does or does not prevent discrimination of the spectrum pattern. It is only shown for the particular types of signals and maskers; however, the same approach may be used for various types.

It has been previously noted [35–37] that the growth of masking of the rippled-spectrum discrimination is different for on- and low-frequency maskers. The data presented herein specify this observation. At signal variation, the low-frequency masker level at threshold varied markedly less than the on-frequency masker level, i.e., the masking growth is higher for low-frequency maskers than for on-frequency maskers. These features of the masking growth resemble those observed in experiments with masking of short-tone detection [39, 40]. In those studies, the difference between the masking growths of on- and off-frequency maskers was considered a manifestation of compressive nonlinearity in the cochlea. The signal and on-frequency masker stimulate the same cochlear region; therefore, they equally involve the active and passive cochlear mechanisms and they are equally susceptible to the compressive nonlinearity of the active mechanism. Alternatively, low-frequency maskers mostly affect the signal representation by the linear passive mechanism, so their masking effect is not influenced by compression. Therefore, the data from the low-frequency masking provide an estimate of the cochlear compression of the signal.

It is reasonable to suppose that the difference between the on- and low-frequency masking of the rippled-spectrum discrimination is of the same nature as for the masking of tone detection, i.e., it reflects the cochlear compressive non-linearity. In the excitation pattern produced within the cochlea, both the on-frequency masking and upward spreading low-frequency masking overlap the signal, reducing the ripple depth and deteriorating the ripple-pattern discrimination. As this occurs, the excitation patterns of the signal and on-frequency masker are subjected to compression, whereas the upward spread of the low-frequency masker is not compressed.

**Fig 7:**
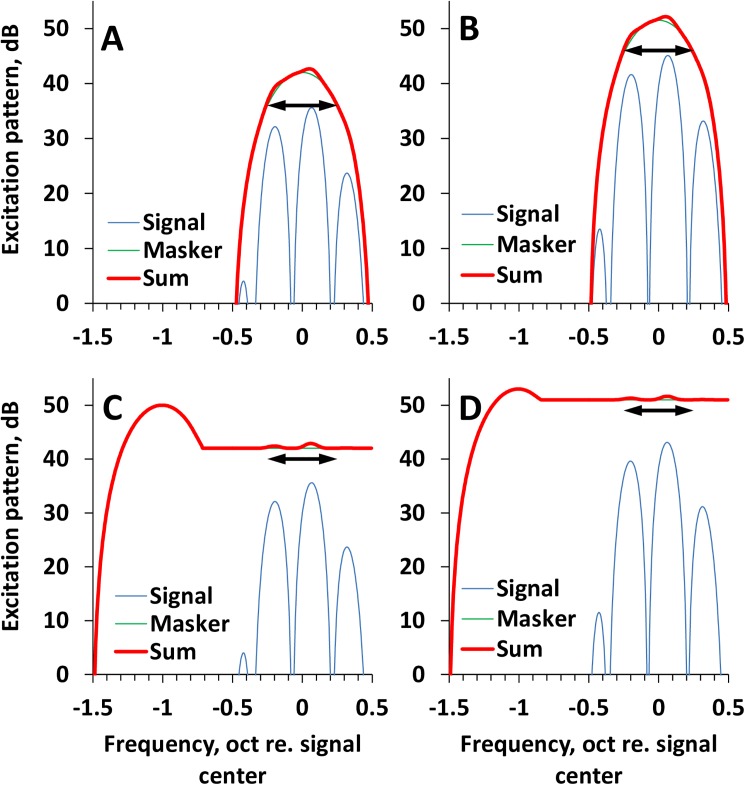
Schematic model of the interaction between signals and maskers. Hypothetical excitation patterns for different combinations of signals and maskers. **A.** 50-dB signal and 56-dB on-frequency masker. **B.** 80-dB signal and 86-dB on-frequency masker. **C.** 50-dD signal and 87-dB low-frequency masker. **D.** 80-dB signal and 96-dB low-frequency masker. Signals and the main lobes of maskers are subjected to compression of 0.3 dB/dB, and the upward-spreading tail is not compressed. On-frequency maskers differing by 30 dB result in equal 1-dB depths of ripples in the sum excitation patterns for both 50-dB and 80-dB signals. Low-frequency maskers that differ by 9 dB produce the same 1-dB depths of ripples in the sum excitation patterns for both 50-db and 80-dB signals. Double-headed arrows depict the range of 1-dB deep ripples in the sum excitation patterns.

These processes are schematically illustrated by a qualitative model in Fig 7, which presents excitation patterns for an arbitrarily chosen compression rate of 0.3 dB/dB above 30 dB SPL. Both 50-dB and 80-dB signals produce excitation patterns that have maxima at 37 and 45 dB, respectively (Fig 7A and 7B). On-frequency maskers exceeding the signals by 6 dB produce excitation patterns with maxima at 43 and 51 dB, respectively. The sum of these two patterns features ripples with a 1-dB depth; 1 dB is a threshold for ripple depth resolution [33]. The low-frequency maskers produce an excitation pattern containing a main lobe and upward spreading “tail”; for simplicity, this tail is assumed to be flat. A masker level increase by 9 dB (C to D) increases the main lobe level by 3 dB; however, the upward spreading tail increases by 9 dB, resulting in 1-dB ripples of the sum pattern for both 50-dB and 80-dB signals.

It is noteworthy that apart from the excitation-pattern model, a temporal processing model may also be applied to analyze ripple pattern discrimination, e.g., [40]. However, for the spectrum patterns used in this study (band-limited signal with frequency-proportional ripples), the temporal processing model is less appropriate than the excitation-pattern model [34].

**Fig 8:**
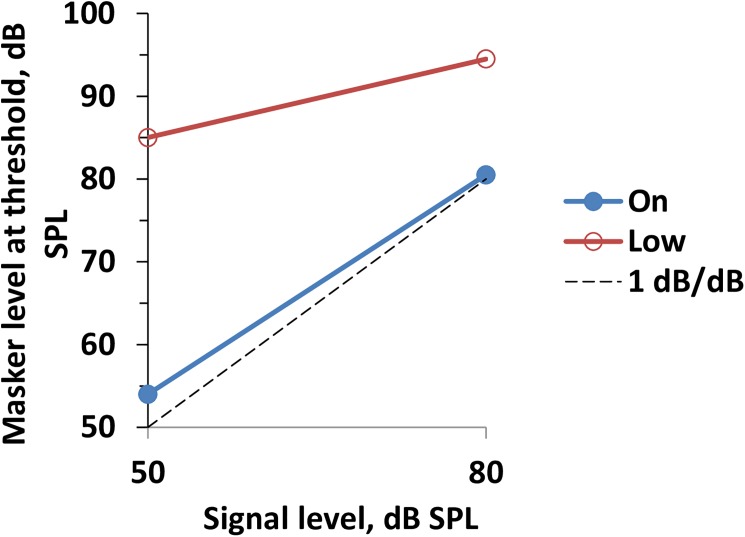
Dependence of the masker level at threshold on the signal level. Data for the on- and low-frequency (–1.25 oct) maskers, as indicated in the legend. Dashed line: 1 dB/dB slope.

Comparison of the on-frequency and low-frequency masking data allows for estimation of the contribution of compressive non-linearity to rippled-spectrum discrimination. From our experiments, only two signal levels, 50 and 80 dB SPL, were available for this estimation; therefore, the data do not allow for detailed tracing of the compression dependence on the level. However, the data at least enable the evaluation of the overall degree of compression within the investigated range. For the –1.25-oct masker, which may be considered a true off-frequency, the signal change of 30 dB resulted in a 9-dB change in the masker level at threshold (Fig 8). Assuming the off-frequency masker effect is linear, this result indicates that the 30-dB signal input range was compressed to a 9-dB cochlear response range, i.e., the output/input ratio was 0.3 dB/dB (3.3-times compression). For comparison, similar data for the on-frequency masker are presented in Fig 8. This function featured a masker level at threshold growth of 27.8 dB (from 56.5 to 84.3 dB SPL) within the same 30-dB signal level range, i.e., the output/input ratio was 0.93 dB/dB, which is only slightly less than 1 dB/dB and is in agreement with the equal influence of the compression on both the signal and masker.

The estimate of the compression rate of 0.3 dB/dB obtained in the present study was less than the estimates of 0.16 to 0.17 dB/dB obtained by [41], however it is close to estimates of 0.2 to 0.4 dB/dB obtained by [42, 43] (the lower the dB/dB ratio, the more the compression). A model by Glasberg and Moore [44] predicts compression of 0.28 dB/dB within the 50–80 dB range at a maximum gain of the active cochlear mechanism of 60 dB. At a maximum gain of the active mechanism of 50 dB, the predicted compression is 0.4 dB/dB. These predictions agree with the obtained compression rate of 0.3 dB/dB.

In the experiments with the masking of the tone detection, several precautions were taken to avoid effects that could distort the results, including lateral suppression and off-frequency listening. To avoid the lateral suppression effect, the forward masking paradigm, rather than simultaneous masking, was used with the assumption that suppression stops almost instantly with the offset of the suppressor [41]. To avoid the off-frequency listening effect, a noise was applied in the expected off-frequency listening region [41] or the signal minimally exceeding the baseline threshold was used [42, 43]. In the latter case, the masking growth was tested by variation of the gap between the masker and signal.

None of the precautions could be taken in our experiments that exploited the rippled-spectrum signals. The forward masking could not be used because of the rather long duration of the signals (see the Methods for motivation of the long signals); therefore, only simultaneous masking was possible. The rather wide spectrum of the rippled-spectrum signals did not allow for depiction of the expected region of the off-frequency listening and application of a noise in this region without affecting the signal itself. Nevertheless, substantial compression was observed. It means that neither off-frequency listening nor lateral suppression, if present, prevented compression in the present study. This difference from the previous tone-detection data originates from the experimental paradigm that required discrimination of the spectra instead of detection of a tone probe:

1. The ripple pattern discrimination was measured within an above-threshold level range. Within this range, the active mechanism dominates over the passive mechanism until its gain is at least several dB; e.g., at a gain as low as 10 dB, the active mechanism transfers 90% of the signal power, i.e., dominates. Therefore, moderate decrease of the gain of the active mechanism due to lateral suppression does not prevent manifestations of compressive nonlinearity.
2. The ripple pattern discrimination was measured within a signal bandwidth that was several times as large as the auditory filter width with a similarly wide masker bandwidth. Within this band, the precise location of the best-response cochlear site should not be crucial.

In summary, it may be stated that the data presented herein indicate how much the cochlear compressive nonlinearity influences the discrimination of spectrum patterns. Because compressive nonlinearity is a basic feature of the auditory system, its contribution to complex spectrum discrimination is anticipated. However, for a complex-spectrum signal, compression is different for different spectral components depending on their frequency and level; as a result, the net effect on discrimination of complex-spectrum signal may be difficult to compute. The data presented herein show the real net effect of compression on the discrimination of complex spectra. It revealed certain peculiarities of this effect. In particular, compression fully manifested in the discrimination of signals in simultaneous low-frequency background noise, which may be an important factor determining signal discrimination in noise.

At the present stage of study, the conclusion concerns a particular representative of complex-spectrum signal and limited range of spectrum parameters. Generalization of the conclusion requires further investigation.
